# Genetic screens in *Saccharomyces cerevisiae* identify a role for 40S ribosome recycling factors Tma20 and Tma22 in nonsense-mediated decay

**DOI:** 10.1093/g3journal/jkad295

**Published:** 2024-01-10

**Authors:** Miguel Pacheco, Karole N D’Orazio, Laura N Lessen, Anthony J Veltri, Zachary Neiman, Raphael Loll-Krippleber, Grant W Brown, Rachel Green

**Affiliations:** Department of Molecular Biology and Genetics, Howard Hughes Medical Institute, Johns Hopkins University School of Medicine, Baltimore, MD 21205, USA; Department of Molecular Biology and Genetics, Howard Hughes Medical Institute, Johns Hopkins University School of Medicine, Baltimore, MD 21205, USA; Department of Molecular Biology and Genetics, Howard Hughes Medical Institute, Johns Hopkins University School of Medicine, Baltimore, MD 21205, USA; Department of Molecular Biology and Genetics, Howard Hughes Medical Institute, Johns Hopkins University School of Medicine, Baltimore, MD 21205, USA; Department of Molecular Biology and Genetics, Howard Hughes Medical Institute, Johns Hopkins University School of Medicine, Baltimore, MD 21205, USA; Department of Biochemistry and Donnelly Centre, University of Toronto, Toronto, ON M5S 3E1, Canada; Department of Biochemistry and Donnelly Centre, University of Toronto, Toronto, ON M5S 3E1, Canada; Department of Molecular Biology and Genetics, Howard Hughes Medical Institute, Johns Hopkins University School of Medicine, Baltimore, MD 21205, USA

**Keywords:** nonsense-mediated decay, ribosome recycling, yeast, translation

## Abstract

The decay of messenger RNA with a premature termination codon by nonsense-mediated decay (NMD) is an important regulatory pathway for eukaryotes and an essential pathway in mammals. NMD is typically triggered by the ribosome terminating at a stop codon that is aberrantly distant from the poly-A tail. Here, we use a fluorescence screen to identify factors involved in NMD in *Saccharomyces cerevisiae*. In addition to the known NMD factors, including the entire UPF family (UPF1, UPF2, and UPF3), as well as *NMD4* and *EBS1*, we identify factors known to function in posttermination recycling and characterize their contribution to NMD. These observations in *S. cerevisiae* expand on data in mammals indicating that the 60S recycling factor ABCE1 is important for NMD by showing that perturbations in factors implicated in 40S recycling also correlate with a loss of NMD.

## Introduction

Nonsense-mediated decay (NMD) is a quality control pathway that targets mRNAs for decay when ribosomes encounter an early or “premature” termination codon (PTC) (Maquat 2004). PTCs can arise from errors in the nucleus such as missplicing events or mutations during DNA replication and transcription, or from errors in translation such as the use of alternative initiation sites or ribosome frameshifting (Popp and Maquat 2013). NMD also plays a broad regulatory role in eukaryotes by targeting both functional, alternatively spliced isoforms, and processed mRNAs that leave the nucleus but that do not encode functional gene products (e.g. long noncoding RNAs) (Smith and Baker 2015). In each scenario, NMD is signaled through ribosome-dependent stop codon recognition and the mRNA is rapidly decayed.

NMD depends on the UPF and SMG-related proteins in all systems (Rebbapragada and Lykke-Andersen 2009; Karousis *et al.* 2016; Kurosaki and Maquat 2016). These factors are critical for the recognition of terminating/recycling ribosomes at PTCs and for triggering the recruitment of RNA decay machinery. Specifically, the NMD-central RNA helicase Upf1 interacts directly with eRF1 and eRF3 (Czaplinski *et al.* 1998; Kobayashi *et al.* 2004), and this interaction is modulated by the phosphorylation status of Upf1 in mammalian systems (Grimson *et al.* 2004; Kashima *et al.* 2006). At the same time, eRF3 interacts with the highly conserved NMD factors Upf2 and Upf3 to stabilize a complex of Upf1, Upf2, and Upf3 (Wang *et al.* 2001). What is essential to understanding the mechanism and specificity of NMD is an understanding of how the ribosome, and in turn release factors and recycling factors, distinguishes between normal termination codon (NTC) and PTC.

The normal processes of translation termination and recycling have been robustly characterized and begin when the ribosome encounters a stop codon (UAA, UAG, or UGA) in the A site. The complex of eRF3:eRF1 recognizes the 3 stop codons and eRF3 then deposits eRF1 into the A site, in a manner analogous to eEF1A loading aminoacyl-tRNAs there during elongation (Hellen 2018; Lawson *et al.* 2021). Following the release of eRF3, the ATPase Rli1 (or ABCE1 in mammals) binds to the ribosome to promote termination (Shoemaker and Green 2011) and ribosome recycling (Pisarev *et al.* 2010; Shoemaker and Green 2011) in which the 60S subunit dissociates from the complex composed of the 40S subunit, mRNA, and P-site tRNA. Hcr1, a loosely bound member of the eIF3 initiation complex, has also been implicated in the recruitment of Rli1 to termination sites and in 80S recycling (Khoshnevis *et al.* 2010; Beznosková *et al.* 2013). In the final steps of recycling, the 40S subunit is dissociated from the tRNA and mRNA in a reaction promoted by 3 proteins known as Tma64, Tma20, and Tma22 in yeast (or eIF2D, MCT-1, and DENR in mammals) (Skabkin *et al.* 2010; Lomakin *et al.* 2017; Weisser *et al.* 2017; Young *et al.* 2018).

During any of the steps of termination and recycling, the activity of the ribosome at the stop codon can in principle signal NMD. What then are the features that distinguish between recognition of NTCs and PTCs that lead to NMD? In broad terms, the “context” of a stop codon within an mRNA determines whether the mRNA is targeted for NMD and could include the following: (1) different nucleotide sequences that affect the recruitment of termination and recycling factors, (2) proximity to the poly-A tail, and (3) the composition and context of local RNA-binding proteins. While much is known about how these different models might dictate NMD, we reasoned that important players in the pathway might remain undiscovered and could shed light on molecular mechanism.

Here, using a bidirectional fluorescent reporter in *Saccharomyces cerevisiae*, we screen for factors that contribute specifically to the decay of the mRNA or its translational repression during NMD. Along with the known NMD regulators in yeast, we identify a group of genes involved in translation termination and recycling. We find that deletion of the known 40S recycling factors *TMA20* and *TMA22* leads to the stabilization of NMD substrates, and, similar to recent results describing the role of the 60S recycling factor ABCE1 in NMD in mammals (Zhu *et al.* 2020), we find that *HCR1* deletion leads to stabilization of NMD substrates. Taken together with published data demonstrating increased ribosome occupancy in 3′UTRs upon loss of Tma20/22/64 (Young *et al.* 2018), these data support a model in which perturbations to ribosome recycling disrupt critical signals for NMD.

## Materials and methods

### Plasmid construction

The OPT reporter plasmid, or pKD065, was constructed as described in D’Orazio *et al.* (2019). The NMD reporter plasmid (pKD081) was constructed by inserting an in-frame “UAA” stop codon 384-bp upstream of the *HIS3*-coding region of pKD065.

### Yeast strains and growth conditions

Yeast strains used in this study are described in Supplementary File 5 and are derivatives of BY4741 unless specified otherwise. Yeast strains were constructed using standard lithium acetate transformations. Reporter strains were constructed by linearizing the given plasmids with StuI and integrating them into the *ADE2* locus of BY4741. Deletion strains were constructed by replacing the gene of interest with drug resistance cassettes at the given locus; see genotypes in Supplementary File 5.

For synthetic genetic array (SGA) experiments, query strains for the deletion screens were constructed by introducing the RFP–GFP-2A-FLAG-HIS3 cassettes from StuI digested pKD065 or pKD081 at the *ADE2* locus in Y7092 (Tong *et al.* 2001; Tong and Boone 2006) (Supplementary File 5).

For gal-induced growths, overnight cultures were grown in YPAGR media (Supplementary File 5). These overnight cultures were then diluted in the same media to an OD of 0.1 and harvested at an OD of 0.4–0.5.

### Flow cytometry

Flow cytometry of individual strains was performed as in D’Orazio *et al.* (2019). Briefly, cells were harvested in log phase and washed with PBS once and then ran on a Millipore Guava easyCyte flow cytometer for GFP and RFP detection using 488- and 532-nm excitation lasers, respectively. Data for 10,000 cells were collected and gated based on size. Flow cytometry was done in triplicate, with each group of cells taken from individual growths. For triplicate plots, the average of each individual flow cytometry sample was taken and normalized to the indicated strain. The log of the fraction was then plotted for each experiment.

### Northern blots

Northern blots were performed as in D’Orazio *et al.* (2019). Briefly, 25 ml of log-phase cells were harvested. RNA was isolated and 5 μg of RNA was loaded into a 1.2% agarose, formaldehyde denaturing gel and run for 1.5 h. The RNA was vacuum transferred to a nitrocellulose membrane (N + H bond, Amersham). The membrane was UV cross-linked, placed in a prehybridization buffer, and rotated at 42°C for an hour. The indicated DNA oligo listed in Supplementary File 5 was 5′ end-labeled using gamma-ATP and T4 Polynucleotide Kinase radioactive labeling protocol from NEB. Labeled oligos were purified using GE Healthcare illustra ProbeQuant G-50 micro columns, and the membrane was probed overnight, rotating at 42°C. The membrane was washed 3 times in 2× SSC and 0.1% SDS for 20 min at 30°C and then exposed to a phosphoscreen. The phosphoscreen was scanned using a Typhoon FLA 9500.

### Western blots

Protein isolation and western blotting were performed as discussed in D’Orazio *et al.* (2019).

### Reporter SGA screens

#### SGA procedure

SGA screens were performed using a Biomatrix Robot with a few modifications (S&P Robotics Inc.). Briefly, yKD176 and yKD179 query strains (Supplementary File 5) were crossed individually with the yeast nonessential gene deletion library (Tong *et al.* 2001). The deletion library was arrayed in a 1536-format with 4 colonies per deletion strain. Because we found that our query strains had a slightly lower mating efficiency and a growth defect, incubation times for every step of the SGA protocol were prolonged by 50–75%. Mating and sporulation steps were performed on standard SGA media (Tong *et al.* 2001; Tong and Boone 2006).

For each deletion screen, diploid strains were selected on DIP media and then haploid double mutant strains were selected for multiple rounds on HAP media listed in Supplementary File 5. Finally, to induce reporter expression, cells were pinned onto haploid double mutant selection medium with raffinose and galactose at a final concentration of 2% (HAPGR media listed in Supplementary File 5). Cells were grown for 26–30 h before scanning on a Typhoon FLA9500 (GE Healthcare) fluorescence scanner equipped with 488- and 532-nm excitation lasers and 520/40 and 610/30 emission filters. Plates were also photographed using a robotic system developed by S&P Robotics Inc. in order to determine colony size.

#### Reporter screen analysis

Screen analysis was performed as described in previous manuscripts (Kainth *et al.* 2009; Hendry *et al.* 2015; D’Orazio *et al.* 2019). Briefly, GFP and RFP fluorescent intensity data were collected using TIGR Spotfinder microarray software (Saeed *et al.* 2003). Colony size data were collected using SGATools (Wagih *et al.* 2013) (http://sgatools.ccbr.utoronto.ca/). After border strains and size outliers (<1,500 or >6,000 pixels) were eliminated, median and mean GFP and RFP values were taken. We then calculated log_2_(mean GFP/mean RFP) for the replicates and performed a LOESS normalization for each plate. Based on this LOESS-normalized value, *Z*-scores were calculated without multiple hypothesis testing correction. Strains for the NMD reporter with a *Z*-score greater than 2.0 or less than −2.0 were considered a hit if their *Z*-score in the OPT reporter was not also an outlier.

#### Venn diagrams

The Venn diagram was created using BioVenn, and the input was deletion strains with a *Z*-score greater than 2.0 or less than −2.0.

#### GO term analysis

Gene ontology (GO) term analysis was performed with the data from the screen using a list of hits that were in the cutoff of *Z* > 2.0 or *Z* < −2.0, and the input gene list is the genes that GFP/RFP data were successfully acquired for in the deletion array screen. The *P*-value cutoff was set to <10^−7^ using GOrilla (Eden *et al.* 2007, 2009). Duplicate strains were removed during analysis.

#### Validation screen

We selected NMD reporter genes that gave a *Z*-score greater than 2.0 or less than −2.0. Then, we removed any hits that also had a *Z*-score greater than 2.0 or less than −2.0 for the OPT reporter. Strains from the haploid deletion collection were subsequently struck out and transformed with the NMD reporter plasmids (pKD081). Three individual colonies from each transformation were isolated. Strains that did not grow were dropped from the experiment, yielding 100 deletion strains to test (Supplementary File 4). These biological replicates were grown overnight in a flow cytometer plate in YPAGR and put on the flow cytometer the next day. The triplicate flow cytometry data for each strain were analyzed, normalizing to the HIS3 controls for all plates tested.

## Results

### Developing a reporter system for nonsense-mediated RNA decay

**Fig. 1.**
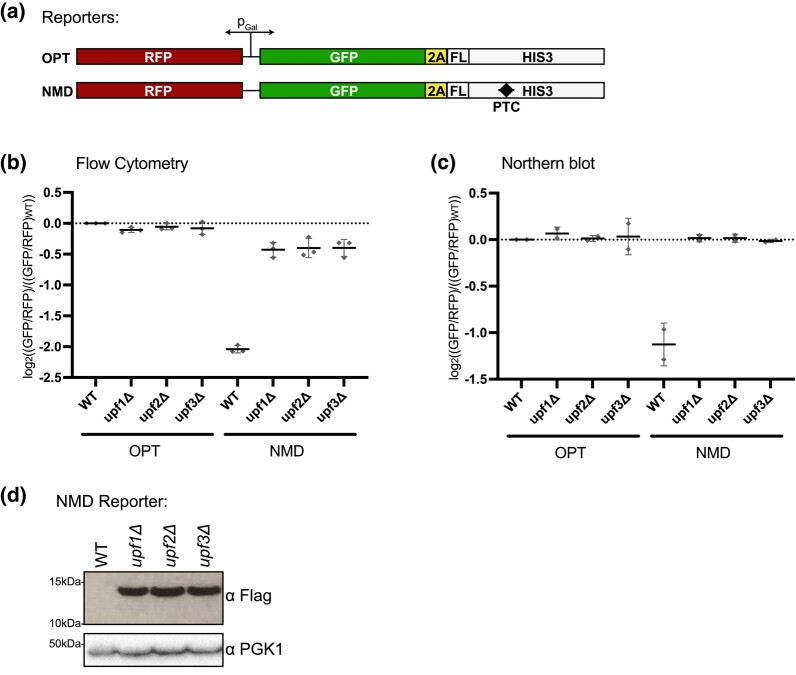
Fluorescent reporters reflect mRNA levels of an NMD target. a) Schematic of reporters with a bidirectional, galactose-inducible promoter. RFP is transcribed in one direction and GFP tethered to His3 via the 2A peptide and FLAG tag (FL) is transcribed in the other direction. b) Individual averages of 3 different flow cytometry experiments performed on the indicated strains with the indicated reporters are shown. The GFP/RFP signal for each given strain normalized to the WT strain with the OPT reporter is plotted. c) Northern blot quantification of 2 unique northern blots is shown. The GFP/RFP signal for each strain, normalized to the WT strain with the OPT reporter, is plotted. d) Western blot of the flag-tagged NMD reporter in the given backgrounds is shown with PGK1 as a control.

To identify genes necessary for NMD in yeast, we designed gene constructs that would report on both mRNA level and translational repression but not on nascent peptide stability (D’Orazio *et al.* 2019). To implement this, we used a GFP-His3-conjugated protein and inserted a viral 2A peptide to effectively dissociate the GFP reporter protein from the downstream His3 protein that encodes a PTC. To control for overall expression, we used a bidirectional, inducible galactose promoter that expressed RFP in the opposite direction from GFP-2A-His3, and we normalized to RFP expression. The GFP reporter construct contains a FLAG epitope at the N terminus of His3 for detection of the peptide downstream of the 2A signal (Fig. 1a). In the His3 ORF, we added a PTC to create an NMD signal (the NMD reporter) while the control reporter (the OPT reporter) contained no PTC (Fig. 1a). As anticipated for an NMD reporter construct, we find that insertion of a PTC 384-bp upstream from the normal stop codon of His3 leads to a 3-fold decrease in GFP/RFP levels as determined by flow cytometry and a 2–3-fold decrease in RNA as determined by northern blot analysis (Fig. 1b and c; representative northern blots shown in Supplementary Fig. 2a).

To confirm that the decreases in RNA levels for the reporter reflect NMD, we deleted the core NMD genes, *UPF1*, *UPF2*, and *UPF3*, and saw a restoration of RNA levels for the NMD reporter by both flow cytometry and northern blots (Fig. 1b and c); as expected, loss of these same factors has no effect on the OPT reporter. Importantly, previous studies have established that NMD impacts the half-life of PTC-containing mRNAs and that deletion of the UPFs results in mRNA stabilization (Leeds *et al.* 1991, 1992; Cui *et al.* 1995; Lee and Culbertson 1995; He *et al.* 1997). These observations lay the groundwork for utilizing GFP output as an indicator for mRNA levels for these reporters in a genetic screen.

Interestingly, in the NMD reporter strains, we saw that GFP RNA levels were fully restored by deletion of *UPF1/2/3* but GFP protein levels were only partially restored (compare Fig. 1b and c). These observations invite speculation about potential translational repression of NMD mRNAs as previously documented (Unterholzner and Izaurralde 2004; Loh *et al.* 2013; Zinshteyn *et al.* 2021). We next utilized the FLAG epitope to look at the peptide product of the NMD reporter (Fig. 1d). In wild-type (WT) cells, the FLAG epitope was undetectable via western blot as the mRNA was degraded by NMD and very little peptide was made. Reassuringly, in the *UPF1/2/3* deletion strains, this peptide construct was stabilized to a similar extent as observed by flow cytometry (Fig. 1d compared with Fig. 1b).

### Screen to identify factors involved in NMD

**Fig. 2.**
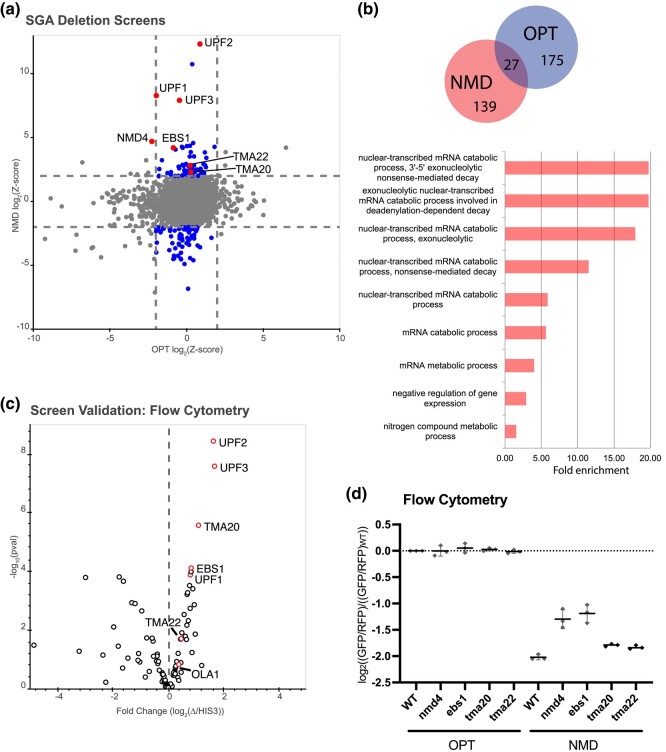
Yeast SGA screens identify genes important for repression of NMD targets. a) Plot of *Z*-scores from OPT reporter deletion screen vs the NMD reporter deletion screen. *Z*-scores reflecting the significance of log_2_(GFP/RFP) values from each deletion strain are plotted against each other for the 2 different GFP reporters. Dashed lines represent cutoffs at a *Z*-score greater than 2 or less than −2 for each reporter. Highlighted dots represent deletion strains that have a *Z*-score value outside the cutoff for the NMD reporter, but not for the OPT reporter. Labeled dots identify the strains known to affect NMD and/or strains of interest in this study. b) (Top) Venn diagram showing the overlap between the OPT screen hits and the NMD screen hits. (Bottom) GO enrichment terms for cellular processes identified from the NMD hits performed using GOrilla software. Note: GO analysis for the OPT reporter hits were insufficient to yield enrichment data. c) Volcano plot showing data from a follow-up screen using newly constructed yeast strains and flow cytometry. The *x*-axis compares the fold change of individual deletion strains to the control *HIS3*-deletion strain. Data for each dot were obtained in triplicate and *P*-values are plotted on the *y*-axis. Red dots identify the strains known to affect NMD and/or strains of interest in this study. d) Individual averages of 3 different flow cytometry experiments performed on the indicated strains with the indicated reporters are shown. The GFP/RFP signal for each given strain normalized to the WT strain with the OPT reporter is plotted.

We next performed a high-throughput reverse genetic screen using the *S. cerevisiae* reporter-SGA (R-SGA) method (Tong *et al.* 2001; Fillingham *et al.* 2009). In R-SGA, the reporter haploid strain is crossed with an array of viable haploid budding yeast deletion strains. Then, using a series of selections, an array of haploid strains containing both the reporter gene and a single gene deletion is produced, allowing reporter activity to be scored in each deletion mutant. We performed 2 independent screens using the OPT reporter (screen 1) and NMD reporter (screen 2) strains, where the reporter/deletion arrays were maintained on glucose media and then the cells were shifted to galactose to induce reporter expression. The GFP and RFP signals from the arrays were evaluated by fluorimetry, yielding a readout for both the OPT and NMD reporter (Supplementary Fig. 1a and b) (D’Orazio *et al.* 2019). For each strain in the plate array, *Z*-score-normalized ratios of GFP intensity over RFP intensity were calculated. In this setting, Z-scores represent the deviation of the GFP/RFP ratio for a given strain from the mean GFP/RFP ratio for the given array; these data for the OPT and NMD reporter strains are plotted against one another in Fig. 2a (raw data are given in Supplementary Files 1–3). From the NMD screen, there were 76 hits with a *Z*-score above 2.0 and 94 hits with a *Z*-score below −2.0 (Fig. 2b; duplicate strains are removed in the Venn diagram). The OPT reporter screen, which has been previously published in D’Orazio *et al.* (2019), also included 64 hits with a *Z*-score above 2.0 and 138 hits with a *Z*-score below −2.0, with 27 genes overlapping as hits in both the OPT and NMD screens that we did not explore further (Fig. 2b) (D’Orazio *et al.* 2019). GO enrichment data show that the hits in the NMD screen are strongly enriched in “mRNA metabolic process” and in “nuclear-transcribed mRNA catabolic process, NMD,” while there were no GO terms for the hits in the OPT screen with a similar level of enrichment (Fig. 2b). Reassuringly, *UPF1/2/3* deletion strains exhibited some of the largest deviations from the mean in the NMD reporter strains, immediately validating the potential of the screen (Fig. 2a). Interestingly, *NMD4* and *EBS1* deletions are also strong candidates that cause an increase in GFP signal for the NMD reporter (Fig. 2a). Nmd4 and Ebs1 are homologs of the Smg6 and Smg5/7 proteins in mammals that are key players in the nucleolytic decay of mammalian NMD substrates (Eberle *et al.* 2009; Lykke-Andersen *et al.* 2014).

To validate our screen, we independently transformed the NMD reporter into strains from the haploid deletion collection corresponding to the NMD screen hits with a *Z*-score less than −2.0 or greater than 2.0. In this list, we excluded the 27 genes that were also hits in the OPT screen, ultimately yielding 139 genes to be validated. Strains that did not grow were dropped from the experiment, leaving 100 strains to be tested (Supplementary File 4). We individually analyzed these 100 newly constructed strains by flow cytometry and plotted the fold change of GFP/RFP for each candidate gene deletion relative to a *HIS3*-deletion control (Fig. 2c and Supplementary File 4). Among the validated hits were genes encoding the UPF proteins, Nmd4, and Ebs1 but also 2 genes encoding factors that were previously implicated in ribosome recycling—*TMA20* and *TMA22* (Skabkin *et al.* 2010; Samanfar *et al.* 2014).

We next decided to focus on several genes previously implicated in ribosome termination and/or recycling. We reconstructed the *TMA20* and *TMA22* deletions along with deletions for the previously known NMD factors *EBS1* and *NMD4* in the WT BY4741 strain carrying the OPT or NMD reporter and used these reconstructed strains for the rest of this study. We began by performing flow cytometry on *nmd4Δ*, *ebs1Δ*, *tma20Δ*, and *tma22Δ* strains in triplicate. As recently reported, deletion of either *NMD4* or *EBS1* modestly stabilizes the NMD reporter (Fig. 2d) (Dehecq *et al.* 2018). Deletion of *TMA20* and *TMA22* even more modestly, but reproducibly, stabilized the NMD reporter relative to the OPT reporter (Fig. 2d), underscoring the capacity of the R-SGA screens to isolate genes with even mild effects on NMD. Although the deletions showed mild effects on NMD, Tma20 and Tma22 have previously been shown to act redundantly, so we reasoned these mild effects might be indicative of a combinatorial role in NMD for this group of proteins (Skabkin *et al.* 2010; Young *et al.* 2018).

### Perturbations to ribosome recycling correlate with inefficient NMD

**Fig. 3.**
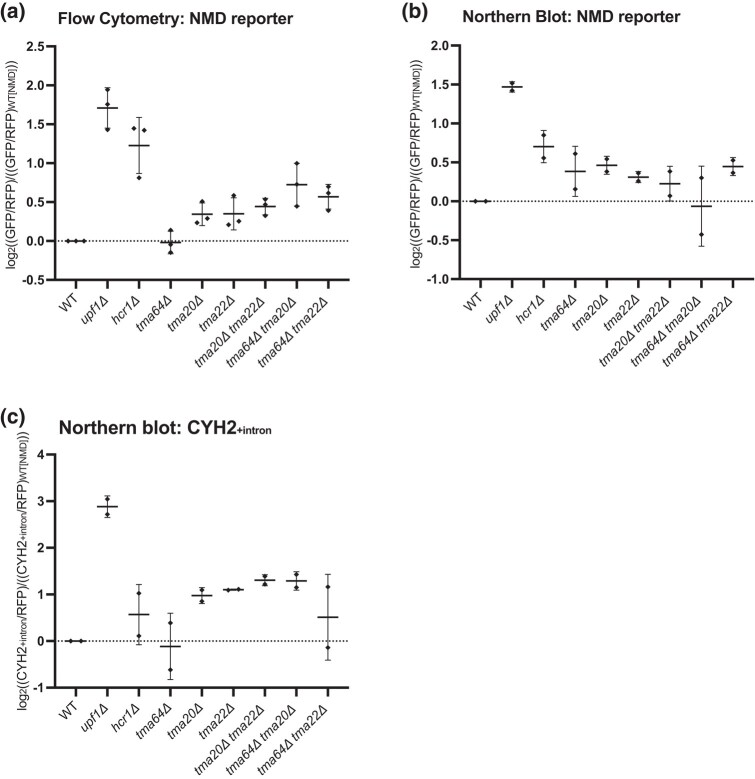
Deletion of 40S and 60S ribosome recycling factors leads to stabilization of exogenous NMD reporter and an endogenous NMD-targeted gene. a) Individual averages of 3 different flow cytometry experiments performed on the indicated strains with the indicated reporters are shown. The GFP/RFP signal for each strain normalized to the WT strain with the OPT reporter is plotted. b, c) Northern blot quantification of 2 unique northern blots is shown for each graph. The (GFP/RFP) signal in b) and the (CYH2^+intron^/RFP) in c) are both normalized to the WT strain with the OPT reporter and plotted.

Ribosome profiling and in vitro biochemical experiments previously showed that Tma20/Tma22 and their mammalian homologs MCT-1/DENR, respectively, are involved in the removal of the 40S subunit from mRNA following Rli1/ABCE1-mediated 60S dissociation (Skabkin *et al.* 2010; Young *et al.* 2018). These factors are homologous to the N- and C-termini of Tma64 whose human homolog, ligatin, functions redundantly with MCT-1 and DENR in in vitro reconstituted systems to release 40S ribosomes and deacylated tRNA from mRNAs following 60S recycling (Skabkin *et al.* 2010). Therefore, we asked if deletion of *TMA64* similarly increases GFP reporter expression as observed for the *tma20Δ* and *tma22Δ* strains. Consistent with the screen data, where *TMA64* did not emerge as a candidate, and with recent data showing only a minor role for Tma64 in vivo (Young *et al.* 2021), deletion of *TMA64* alone did not result in an increase in GFP expression from the NMD reporter (Fig. 3a). As a matter of routine, we made the double deletions of *TMA64* in combination with deletion of *TMA20* or *TMA22*, though the modest increase the GFP signal from the NMD reporter relative to each of the single deletions was not statistically significant (Fig. 3a). Northern blot data for the NMD reporter similarly revealed a modest (but statistically insignificant) stabilization of the NMD reporter mRNA for the *TMA20*, *TMA22*, and *TMA64* deletion strains, while the double deletions (i.e. *tma20Δ tma64Δ*) did not show enhanced effects (Fig. 3b; representative northern blots shown in Supplementary Fig. 2b).

Because *TMA20*, *TMA22*, and *TMA64* deletions have been shown to function in 40S ribosome recycling, we next asked if NMD was also affected by disrupting 60S ribosome recycling by deleting the Rli1 accessory factor gene *HCR1*. While the *hcr1Δ* strain did not emerge from our initial screen due to its slow-growth phenotype, this deletion indeed increased GFP expression and stabilized the NMD reporter (Fig. 3a and b). These data together support the hypothesis that deficiencies in either 40S or 60S ribosome recycling can disrupt NMD.

As final validation of a role for these genes in NMD, we evaluated their effects on a well-characterized endogenous target of NMD, the rare intron-retaining transcript of *CYH2* (He *et al.* 1993), by northern blot analysis. The absence of the 40S recycling factors Tma20 and Tma22 alone again reproducibly stabilized the *CYH2*^*+intron*^ transcript (Fig. 3c; representative northern blots shown in Supplementary Fig. 2b). Deletion of the 60S recycling factor *HCR1*, however, did not have as pronounced an effect on NMD of the intron-containing *CYH2* transcript as it did in flow and northern analyses of the NMD reporter. These data provide broad support for both the 40S and 60S recycling factors playing roles in promoting efficient NMD.

## Discussion

Translation termination and recycling are inherently linked to NMD signaling through the recognition of a premature stop codon by the ribosome. Many previous studies have worked to define how these ribosome activities, termination and recycling, might be connected to the specification of NMD either directly or through interaction with a host of factors potentially bound to the 3′UTR of the mRNA (Czaplinski *et al.* 1998; González *et al.* 2000; Lykke-Andersen *et al.* 2000; Le Hir *et al.* 2001; Wang *et al.* 2001; Kobayashi *et al.* 2004; Hogg and Goff 2010). Furthermore, there are indications that key NMD factors such as Upf1 may directly influence ribosome recycling (Ghosh *et al.* 2010).

Here, we used a newly developed genetic screen in yeast with fluorescent reporter constructs to identify key factors that contribute to NMD. We identify 40S ribosome recycling factors Tma20 and Tma22 in the screen and additionally 60S recycling factor Hcr1 in follow-up experiments, as important modulators of NMD for both reporter constructs and an endogenous NMD target (Figs. 2 and 3). These observations are consistent with earlier studies showing that ribosome reinitiation after a PTC abrogates NMD (Ruiz-Echevarria and Peltz 1996; Zhang and Maquat 1997) and several recent studies in mammals implicating ribosome recycling and 3′UTR ribosome activity in NMD specification (Annibaldis *et al.* 2020; Zhu *et al.* 2020). These data are broadly consistent with NMD models implicating 3′UTR-bound proteins as key determinants (Peltz *et al.* 1993; González *et al.* 2000; Lykke-Andersen *et al.* 2000; Le Hir *et al.* 2001; Hogg and Goff 2010).

Rli1, or ABCE1 in mammals, works with partner protein Hcr1 to stimulate termination and recycle 60S subunits (Pisarev *et al.* 2010; Shoemaker and Green 2011; Beznosková *et al.* 2013; Young and Guydosh 2019). Our data show that deletion of *HCR1* stabilizes NMD substrates (Fig. 3), consistent with recent work done in mammalian cells demonstrating that a loss of ABCE1 stabilizes NMD substrates (Annibaldis *et al.* 2020; Zhu *et al.* 2020). Similarly, we found that deletion of 40S ribosome recycling factors, *TMA64*, *TMA20*, and *TMA22*, also stabilizes NMD substrates (Fig. 3). Ribosome profiling experiments in Rli1 depletion, *hcr1Δ*, *tma64Δtma20Δ*, and *tma64Δtma22Δ* strains, reported a transcriptome-wide increase in the abundance of ribosomes in 3′UTRs (Beznosková *et al.* 2013; Young *et al.* 2018; Young and Guydosh 2019).

Previous studies have established that the 3′UTR is a critical regulator of NMD. In early studies in yeast, specific “downstream elements” (DSEs) were demonstrated to be important for triggering NMD and these DSEs were later shown to be bound by the RNA-binding protein Hrp1 that was critical for NMD for a subset of DSE-containing targets (Peltz *et al.* 1993; González *et al.* 2000). In mammalian cells, the presence of exon junction complexes (EJCs) and the accumulation of Upf1 in the 3′UTR have both been strongly implicated in promoting NMD (Lykke-Andersen *et al.* 2000; Le Hir *et al.* 2001; Hogg and Goff 2010). Taken together, these studies lead to simple models invoking positive contributions to NMD by proximal bound proteins (Upf1 and the EJC) that recruit other critical machinery involved in mRNA decay (such as the SMG proteins). The contributions of such proteins in the 3′UTR to NMD signaling provide 1 potential explanation for our results wherein the presence of scanning and/or translating ribosomes in the 3′UTR disrupts NMD. Alternatively, inefficient ribosome clearing at stop codons may interfere with NMD by perturbing other key steps such as recruitment of deadenylation or decapping machinery or by indirectly impairing translation initiation. Further experiments will clearly be needed to define the mechanism by which loss of Tma20 and Tma22 contributes to impaired targeting of NMD substrates for decay.

## Supplementary Material

jkad295_Supplementary_Data

## Data Availability

Strains and plasmids are available upon request. The supplementary files contain complete lists of all data and analysis from the screen, including raw data from the synthetic genetic array for the OPT and NMD reporter (Supplementary Files 1 and 2, respectively; a condensed spreadsheet with *Z*-scores from both reporters is provided in Supplementary File 3), flow cytometry data for each candidate gene deletion relative to a *HIS3*-deletion control (Supplementary File 4), and a list of yeast strains, plasmids, and DNA oligos used in this study (Supplementary File 5). Supplemental material available at G3 online.
