# Supplementary material for: Genetic screens in Saccharomyces cerevisiae identify a role for 40S ribosome recycling factors Tma20 and Tma22 in nonsense-mediated decay
Source: G3 (Bethesda). 2024 Jan 10;14(3):jkad295. doi: 10.1093/g3journal/jkad295 (PMC10917514; doi:10.1093/g3journal/jkad295)
Supplement: jkad295_Supplementary_Data [file jkad295_supplementary_data.zip › Supplemental_Figure_Legends_G3-2023-404709.docx]

**Figure S1:** Example of plate images from Typhoon scans of the galactose SGA plates. (A and B) Images are scanned using GFP filters (A) and RFP filters (B) for the same exact SGA plate. The arrow points to the *UPF2* deletion colonies for the two different fluorescent scans.

**Figure S2:** Representative northern blot images. (A) shows GFP and RFP northern blots quantified in Main Figure 1C. (B) shows GFP, RFP, and CYH2 northern blots quantified in Main Figure 3B and C.
