# Supplementary figures and images for: Genetic screens in Saccharomyces cerevisiae identify a role for 40S ribosome recycling factors Tma20 and Tma22 in nonsense-mediated decay
Source: G3 (Bethesda). 2024 Jan 10;14(3):jkad295. doi: 10.1093/g3journal/jkad295 (PMC10917514; doi:10.1093/g3journal/jkad295)

**A**

**GFP scan**

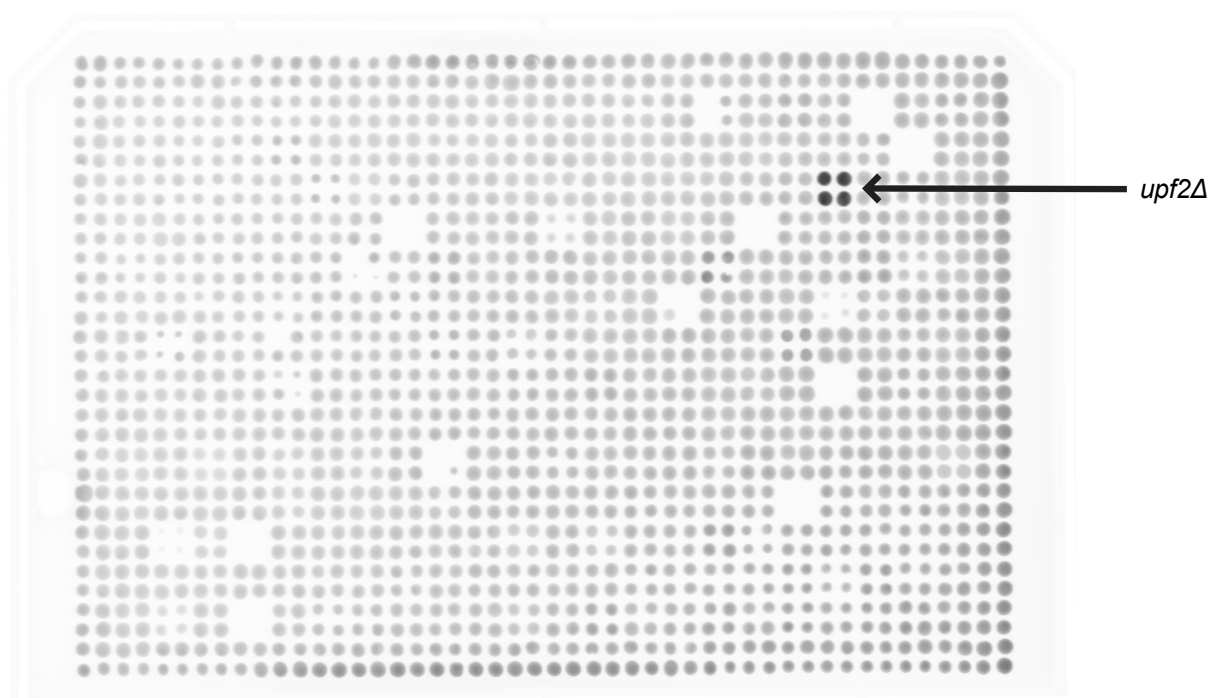

**B**

**RFP scan**

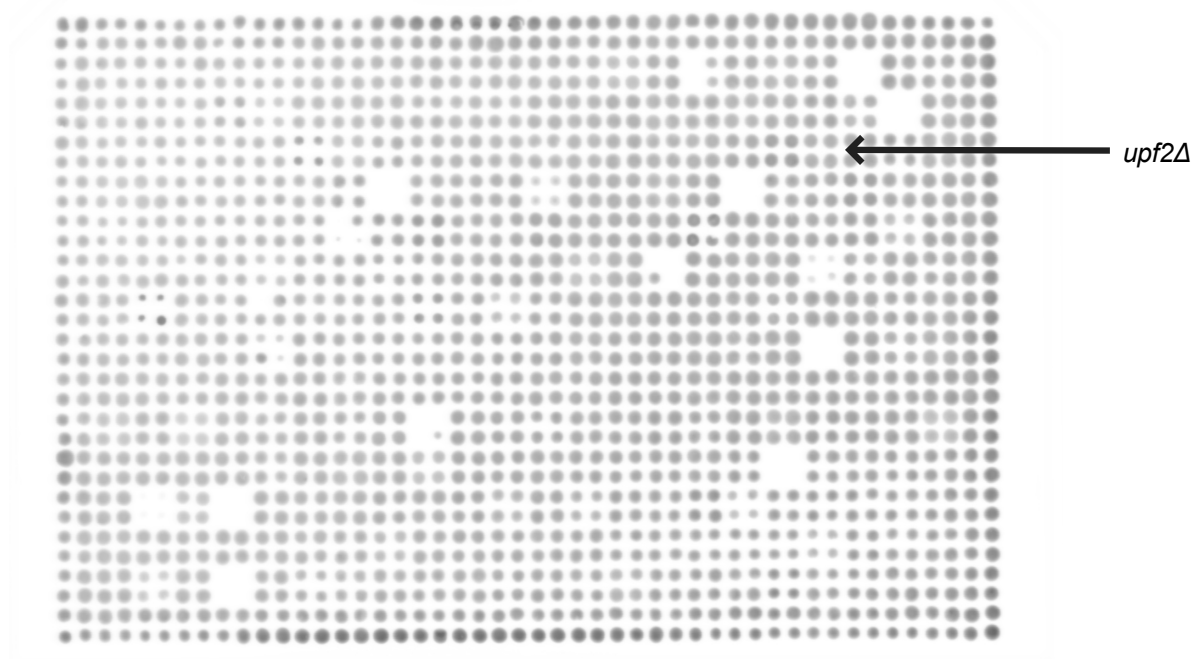

Supplement: jkad295_Supplementary_Data [file jkad295_supplementary_data.zip › Figure_S1_G3-2023-404709.pdf]

**A** Reporter validation: Northern blot

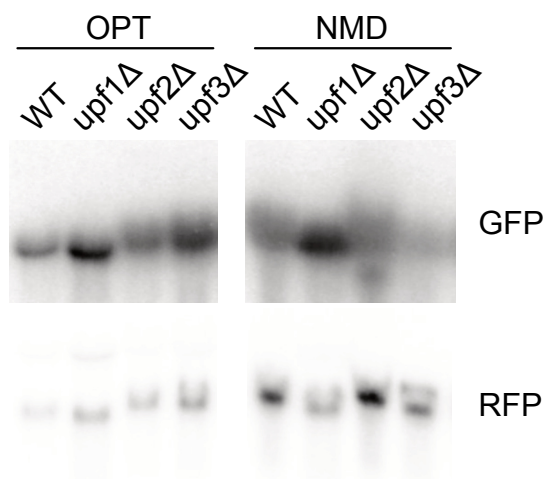

**B** Screen hits validation: Northern blot

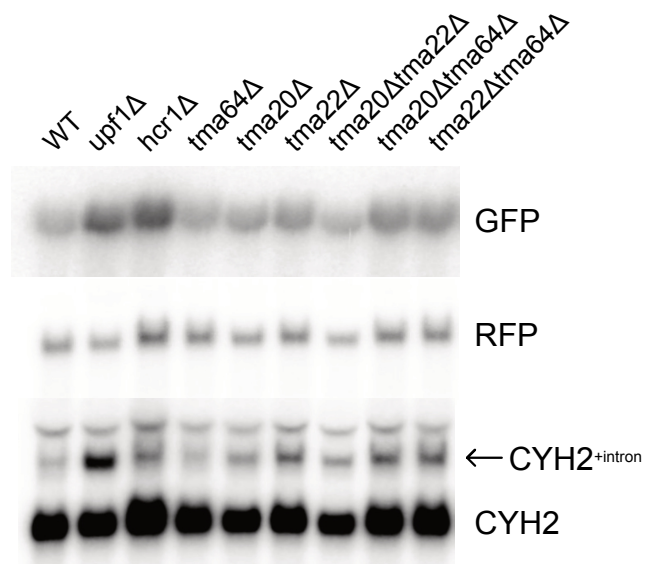

Supplement: jkad295_Supplementary_Data [file jkad295_supplementary_data.zip › Figure_S2_G3-2023-404709.pdf]
